# Family status and women’s career mobility during urban China’s economic transition

**DOI:** 10.4054/demres.2021.44.8

**Published:** 2021-02-02

**Authors:** Guangye He, Xiaogang Wu

**Affiliations:** 1 Department of Sociology, School of Social and Behavior Sciences, Nanjing University, Nanjing, China.; 2 Center for Applied Social and Economic Research, NYU Shanghai, Shanghai, China, and Department of Sociology, New York University, USA.

## Abstract

**BACKGROUND:**

In contrast to the historical experience of Western welfare states, where social and family policies help create more integrated public–private spheres, marketization in China has presented a case of sphere separation. This phenomenon has important implications for the dynamics of gender inequality in economic transition.

**OBJECTIVE:**

This article examines how family status is associated with women’s career mobility in reform-era urban China and the impact of family on women’s career choices across different reform stages.

**METHOD:**

Based on retrospective data from the Chinese General Social Survey (CGSS) in 2008, we adopt discrete-time logit models to examine the effects of marriage and childbearing on women’s upward mobility, the risk of labor market exit, and how the effects vary over time.

**RESULTS:**

Chinese women in the workforce are adversely affected by marriage and having dependent children. They are more likely than men to experience (involuntary, in particular) job exit to fulfill their roles as wives and mothers and less likely to move up in the career ladder. This pattern is more prominent as the economic reform proceeds.

**CONCLUSION:**

Marketization has adversely affected Chinese women’s career outcomes by increasing work–family tension after the work unit (*danwei*) system and socialist programs that supported working women were scrapped.

**CONTRIBUTION:**

This study is one of the few empirical studies to attempt to explain the widening gender gap in China’s job market from the perspective of family using the two-sphere separation framework. The framework originated in Western family studies but has been adapted to suit the context of urban China.

## 1. Introduction

Women are typically responsible for a greater share of household duties than men. Marriage and parenthood tend to exacerbate the traditional gendered division of labor within the family (Bianchi et al. 2000; Bianchi and Milkie 2010; Gupta 1999). To fulfill their family responsibilities, women spend more time and effort on housework and childcare. Some may even have to withdraw from the workforce at certain life stages. Such career interruptions limit women’s promotion chances and higher earnings attainment (Becker 1991).

While women’s reproductive role (i.e., marriage and childbirth) is universal, how it is associated with their career outcomes varies across societies with different institutional arrangements and welfare systems (e.g., Cotter, Hermsen, and Vanneman 1999; Esping-Andersen 1999; Gornick and Meyers 2003; Hegewisch and Gornick 2011; Kolberg and Esping-Andersen 1991; Mandel and Semyonov 2006; Orloff 2002). For example, to promote gender equality, governments in Western countries often institute certain social and family policies, such as paid parental leave and subsidized childcare, to ease women’s role conflict between paid work and unpaid household labor; they may also enact laws and regulations to prohibit open discrimination against women in the labor market (e.g., Beller 1982; Cheng 2016; Gornick and Jacobs 1998; Mandel and Semyonov 2006; Orloff 1993; Van der Lippe and Van Dijk 2002). In pre-reform China, such policies or regulations were implemented through the socialist work unit (*danwei*), a unique and multifunctional institution that provided members with various nonpecuniary benefits and offered social services, such as childcare, to support women’s reproductive role (Bian 1994; Walder 1986, 1992). Since the market reform began in 1978, especially after the mid-1990s, state-owned work units have scrapped many of their social service provisions, and the reproductive burden has been shifted back to families. As a result, work–family conflicts have escalated in urban China, particularly for married women (Ji et al. 2017).

While numerous cross-national studies have examined gender inequality under various welfare regimes (e.g., Esping-Anderson 1999; Hobson 2006; Mandel 2010; Orloff 1996), few have investigated how the impact of marriage and childbirth on women’s labor market chances would be affected by welfare regime changes. The transition from a socialist redistributive economy to a market economy in China led to the reconfiguration of institutional arrangements between work and family (He and Wu 2017; Ji and Wu 2018). The setting thus provides a unique chance for scholars to examine the impact of such reconfigurations on the negative effect of women’s reproductive responsibilities on career advancement in the labor market. Based on retrospective data from the Chinese General Social Survey (CGSS) in 2008 (Bian and Li 2012), we employ discrete-time logit model in event history analysis to investigate how marriage and parenthood would affect women’s career mobility and how such effects differ between men and women across different reform stages.

## 2. Family, work, and women’s career contingency: A two-sphere separation perspective

To illustrate the changing work–family dynamics for women, we employ a conceptual framework of two-sphere separation in Western family studies with certain modifications to suit the context of China (Ji et al. 2017). The conventional research on families and gender roles treats families more or less as closed units (i.e., the private sphere) with prevailing social relations that are fundamentally different from those in other political or economic institutions (i.e., the public sphere) (Ferree 1990). Such a bifurcated view of families and work tends to legitimize gender role differentiation (e.g., Connell 1985; Lopata and Thorne 1978) in which women assume the distinct and primary role in domestic work (Adams 2011), while men serve as the family breadwinners. Women are excluded from participating in the labor market, rendering their subordination to men inevitable (Edwards 2001).

The bifurcated framework of separate spheres, stemming from the context of early industrialization in the West, has been criticized by historians and feminist theorists since the mid-20^th^ century. According to this view, families are fully integrated into wider systems of economic and political institutions, thus members within families may have diverging and sometimes conflicting interests (Ferree 1990). Moreover, women’s increasing presence in labor markets since World War II (Costa 2000; Fullerton 1999; Goldin 1990; Hout and Diprete 2006; Juhn and Potter 2006; Reskin 1993; Shavit and Blossfeld 1993) seems to have further challenged the dichotomous framework of sphere separation in understanding gender inequality in the contemporary world.

In addition to their growing presence in the workforce, women in most developed countries have reached parity with or even exceeded men in educational attainment, yet the gender pay gap persists (Hausmann, Tyson, and Zahidi 2009). Many studies attribute the gender disparities in hiring, promotion, and earnings to different family roles between men and women (Blau and Kahn 2007; Fernandez and Friedrich 2011; Sandberg 2013; Slaughter 2012; Stone 2007). As women are responsible for a greater share of domestic work than men, especially when they have young children to care for, they are seen as being less committed to the workplace and more likely to quit jobs for family reasons (Cha 2010, 2013; Jacobs 1989; Kan and He 2018; Marini 1989; Stone 2007; Treiman and Hartmann 1981). If employed, they are more likely to remain in lower-paid occupations traditionally dominated by women (e.g., He and Wu 2019; He and Zhou 2018; Levanon and Grusky 2016; Marini 1989). Hence, it is women’s family role that holds off their career advancement in the labor markets. Such work–family conflicts demonstrate the dynamic interaction between the public (work) and private (family) spheres.

Although the static view of the two-sphere separation has been challenged, we argue that the bifurcated framework, if adapted to incorporate historical specifics and dynamic perspectives, can still provide a useful framework to gain a deep understanding of working women’s struggles and attempt to achieve work–life balance. While their participation in the labor market may have brought women more bargaining power within families, progress toward gender parity has been stalled due to their unequal burden of housework and childcare. Progress may require an increase in men’s participation in housework and childcare, governmental provision of childcare, and employer policies that assist women in combining their careers with family responsibilities (England, Levine, and Mishel 2020).

Indeed, evidence from comparative studies has shown that government support for paid leave and free childcare are positively associated with women’s full-time employment (Mandel and Semyonov 2006). Studies have also shown that when parental leave is granted to men, they perform more domestic work (Budig, Misra, and Boeckmann 2012). In Nordic countries, family policies that consider women’s labor market participation and men’s childcare involvement are instrumental in promoting gender equality (Guo and Xiao 2013). These studies, nevertheless, are mostly based on the historical experience of Western countries, where the institutional links between family and work have remained relatively stable over time. In addition, public policies are targeted to improve the arrangement of housework and childcare for women, something that was once considered a private matter negotiated between spouses or within families (He and Wu 2019). Rarely do public policies move in the opposite direction. We term such change as the reseparation of public and private spheres. China’s transition to a market economy has provided a unique historical setting in which we examine the implications of such reseparation on women’s work–life balance and gender inequality in the labor market.

## 3. Work units, marketization, and women’s changing work–family balance in China: Research hypotheses

We adopt a historical and dynamic perspective on the public–private sphere separation and situate the changing work–family conflict in the context of China’s transition from a state economy to a market economy (Ji et al. 2017). The transformation of the socialist work organization (known as the work unit or *danwei*) played a pivotal role in the process of the separation or reseparation between the public and private spheres and in enlarging gender inequality in the labor market during the reform era.

Since the founding of the People’s Republic of China, the Communist Party has made a great effort to promote social egalitarianism, including gender egalitarianism. According to Friedrich Engels (1978 [1891]: 744), the premise for women’s emancipation is “the reintroduction of the entire female sex into public industry.” As such, the Communist Party state always encouraged women to participate in socialist production (Whyte and Parish 1984). From the perspective of two-sphere separation, women’s progress into the public sphere would necessarily create a tension with their reproductive role in private family life, as observed in many industrialized societies.

Similar to welfare states in Western countries, the Chinese government had instituted regulations and social policies to alleviate the tension between women’s productive and reproductive roles. For example, the People’s Republic of China Labor Insurance Regulations, instituted in 1951 and further amended in 1953, stipulated for the first time in Chinese history that all female employees enjoyed a 56-day maternity leave with full pay and a 30-day leave with full pay for a miscarriage.^3^ Employers also paid all expenses for checkups during pregnancy and delivery (State Administration Council of People’s Republic of China 1951). In the following year, the Regulations for Kindergartens (draft) called for the government at different levels and employers to develop nurseries and kindergartens, with the aim of reducing “the burden of childcare on mothers so they will have time to participate in political life, productive work, and cultural and educational activities” (Government Administration Council of the Central People’s Government 1951).

As the highly centralized planned economy was quickly installed in the first half of the 1950s, these state regulations and social policies were implemented mainly through publicly owned work units (danwei). For example, in the Joint Notice on Nurseries and Kindergartens issued in 1956, the government provided further guidelines for childcare programs and stipulated that all danwei units be responsible for program expenses, including housing and staffing, as well as the daily administration of nurseries and kindergartens. Moreover, danwei not only offered working women job security and prohibited gender discrimination in pay associated with their domestic roles but also provided various kinds of daily life services (such as canteen service in living quarters) to socialize women’s domestic duties that they would otherwise need to perform within their own families (Walder 1986, 1992; Wu 2002).

In the perspective of the two-sphere framework, some scholars argued that the gendered division of household work was woven into the socialist production process (Song 2011). It was through the unique danwei system that the private sphere and public sphere became more integrated, alleviating the tension between work and family to support women’s employment. Consequently, in the late 1950s and 1960s, more than 90% of urban married women were employed, and the rate of female labor force participation remained high until the early 1990s; gender discrimination was strictly prohibited during the socialist era (Wolf 1985; Zuo and Bian 2001). Studies also found that gender earnings inequality was negligible in the public sector, even in the reform era (He and Wu 2017).

While the state may have been more effective in promoting gender equality in the public sphere through the danwei system, its penetration into the private sphere seems to be less effective, as the change of gendered division of labor within families required substantial cultural changes, even in the Western world (England, Levine, and Mishel 2020). In China, long influenced by a Confucian patriarchal culture, the new communist gender ideology suppressed the traditional gender roles in the private sphere but did not resolve working women’s household responsibilities (Ji et al. 2017; Wu, Ye, and He 2014). After the danwei system was weakened, the retaining gendered division of domestic labor would trap women in work–family conflicts.

By the late 1990s, the market-oriented economic reform fundamentally undermined the danwei system (Wu 2019). The state had attempted to convert danwei into more profit-oriented entities that were less dependent on administrative fiats. Danwei gained more autonomy in the recruitment, remuneration, and dismissal of employees than before. The lifelong employment system with cradle-to-grave welfare in danwei ended (Wu 2010). Meanwhile, the fast-growing private sector has become the most dynamic part of the Chinese economy. Unlike their counterparts in the state sector, these private firms emphasize economic efficiency over social justice when recruiting employees. There is growing evidence of gender discrimination (Parish and Busse 2000), as employers anticipate that women will be less committed to work, especially after getting married and having children (Cao and Hu 2007; He and Wu 2017). As a result of the fundamental institutional changes, the rate of labor force participation in urban China dropped from 89.4% in 1990 to 63.5% in 2005 for women ages 21 to 50 (Wu and Zhou 2015); the earnings ratio between females and males has declined from 86.3% in 1988 to 76.2% in 2004 (Zhang, Hannum, and Wang 2008). Studies have shown that marketization is the main culprit behind these trends (He and Wu 2018).

The transformation of danwei also led to the separation of once-integrated public and private spheres under socialist production and the reconfiguration of work–family arrangements in the new era. Similar to their counterparts in other East Asian countries where marriage is nearly universal (Raymo et al. 2015), most Chinese women are expected to get married, give birth, and prioritize their family obligations over career development (Cheng 2018; Wu, Ye, and He 2014). With the decline of communist gender ideology once backed by the socialist danwei system, traditional patriarchal values have shown signs of revival (Ji et al. 2017). Moreover, since the market reform, numerous publicly funded childcare facilities were scaled down or transformed into fee-paying programs. Danwei no longer provides nursery care to children of ages 0 to 2 and has cut support for preschool childcare. By the year 2006, the number of publicly funded kindergartens had decreased to about one-third of that in 1997. During the same period, private kindergartens had increased from 13.5% to 57.8% of all childcare programs (Ministry of Education 2007).

What do the collapse of the danwei system and the loss of state’s protection mean for women’s careers? In the two-sphere framework, we see the changes as an increase in separation of the private and public spheres and an escalation of work–family conflicts that leave all childcare to the family, particularly to women (Cook and Dong 2011; Zuo and Jiang 2009). These circumstances only exacerbate the disadvantage to women in the labor market (Hughes and Maurer-Fazio 2002; Ji et al. 2017; Qian and Jin 2018; Sun and Chen 2017; Zhao and Hannum 2019). By adopting this conceptual framework, we provide a dynamic perspective on the negative impact of marriage and childbearing on women’s careers in midst of China’s market transition.

The career outcomes of our interest here include upward job mobility and job exit. In the two-sphere framework, given the gendered roles in the public and private spheres, women still take on more family responsibilities than men. Therefore, we expect that marriage may prevent career advancement for women only. For the same reasons, they are also more likely than men to experience job exit, especially involuntary job exit (e.g., layoffs/job loss or family-oriented job exit). We thus propose the following hypotheses to test:

*Hypothesis 1a*: Married women are less likely to achieve upward job mobility than unmarried women, whereas this is not the case for men.
*Hypothesis 1b*: Married women are more likely to experience job exit – in particular, involuntary job exit – than unmarried women, whereas this is not the case for men.

Having children, especially preschool children, may further intensify the traditional gendered division of labor and affect women’s chances of developing their own careers (Mu and Xie 2016; Wu, Ye, and He 2014; Zhang and Hannum 2015; Zhang, Hannum, and Wang 2008; Zuo and Bian 2001). In this vein, we propose the following hypotheses:

*Hypothesis 2a*: Having dependent children reduces women’s upward job mobility, particularly for married women.
*Hypothesis 2b*: Having dependent children increases women’s likelihood of withdrawing from the labor force, particularly for married women.

The gradual dismantling of the danwei system that led to the separation of the private and public spheres since the reform increased work–family conflict for Chinese women, especially for married women with preschool children. These circumstances left them more vulnerable in labor markets. Hence, we further propose the following testable hypotheses:

*Hypothesis 3a*: The effects of marriage on women’s upward job mobility and job exit, especially involuntary job exit, are more prominent in the late reform stage than before.
*Hypothesis 3b*: The effects of having dependent children on women’s upward job mobility and job exit, especially involuntary job exit, are more prominent in the late reform period than before.

While the first two sets (Hypotheses 1a, 1b, 2a, and 2b) pertain to marriage and motherhood penalties on women’s careers and are generally applicable to other societies (see similar findings from China by Cao and Hu 2007; Mu and Xie 2016; Sun and Chen 2017; Zhang and Hannum 2015), the third set aims to empirically verify our key argument about how the impact of marriage and motherhood on women’s careers has changed during the market transition in urban China.

## 4. Data, variables, measures, and methods

### 4.1 Data

This analysis is based on data from the Chinese General Social Survey (CGSS) conducted in 2008. Adopting the multistage stratified random sampling method, CGSS is a nationally representative survey conducted annually and biennially since 2003. The 2008 survey gathered retrospective data on respondents’ life histories, including family composition, marriage, education, and employment. The data is ideal for investigating the issues to be addressed in this article. A total of 6,000 interviews were conducted and completed, with 3,982 from urban areas and 2,018 from rural areas. The response rate is 54.3% (Bian and Li 2012). Since the occupations in rural areas are homogeneous and job mobility is low, we restrict the analysis to the urban sample. After deleting those who have never worked or who have no work record, we are left with 3,028 individuals in the analytical sample.

### 4.2 Variables and measures

There are two dependent variables in this study: whether one has experienced upward mobility and whether one has exited from the labor force. We define upward mobility based on job characteristics, such as promotion within professional or administrative ranks, or an increase in occupational status measured by the International Socio-Economic Index (ISEI; Ganzeboom and Treiman 1996). A labor market exit was recorded if an individual reported having had a job at one point but was no longer working in the subsequent time period. Based on detailed work histories, we further differentiate types of job exit. This variable contains three categories. If respondents reported having had a job in a work unit at any time, we coded it as 1 (being employed). If respondents reported being laid off, experiencing job loss, or being off for family reasons, we coded it as 2 (involuntary job exit). And if respondents reported being self-employed with no work unit, retired, or engaged in farm work at one point in time but no longer, we coded it as 3 (voluntary job exit).^4^

Family status includes marriage and having dependent children (ages 0 to 6). As the survey collected information on both marital status and the first marriage year, we construct marital status as a time-variant covariate (1 if married and 0 otherwise). Presence of dependent children, based on children’s ages in correspondence with respondents’ employment, is also a time-variant covariate. This variable is coded as 0 before a child was born and after the child reached 6 years old.

We include education, party membership, and rural *hukou* status as controls since they are all important factors in career mobility in urban China (e.g., Walder, Li, and Treiman 2000; Wu 2002; Wu and Treiman 2007). These variables are all time-variant covariates. Education is a four-category variable (1 = primary school or below; 2 = junior high school; 3 = senior high school; and 4 = college or more). Both party membership and rural hukou status are binary variables (1 if yes and 0 otherwise). To take into account the baseline occupation, we also include the ISEI of respondents’ first jobs.

To demonstrate the varying job shift patterns across economic sectors, we further control for time-variant work sectors (1 if public sector and 0 otherwise). We code government/party agencies, public institutions, state-owned enterprises, and collective enterprises as the public sector and the rest as the nonpublic sector (He and Wu 2018; Zhou, Tuma, and Moen 1997). To reveal the variations over time, we group the year into three reform stages: the early stage (1978‒1992), when the reform started in rural areas and gradually shifted to urban areas but fundamental institutions remained intact; the middle stage (1993‒1998), following Deng’s southern tour in 1992 and call for further market reform to build a socialist market economy (Naughton 2006); and finally, the late stage (1999‒2008), when state-owned enterprises underwent substantial restructuring and radical privatization (Xu and Wu 2021; Yeh, Yang, and Wang 2015).

### 4.3 Methods and analytical strategy

We employ discrete-time logit models in event history analysis to investigate the dynamic relationship between marriage, childbirth, and job change, given that the time of upward mobility and job exit is measured discretely (Allison 2014). Here, we treat upward mobility as repeated events, as an individual may experience upward mobility more than once. For job exit, we first present the results without differentiating types of job exit. We then adopt competing risk models and estimate job exit with three outcomes to reveal how family roles are associated with men and women’s involuntary job exit across different reform stages.

To conduct event history analysis, we restructure individual-level data into person-year data. The risk set consists of those individuals who have ever held a job. The clock starts at the time when individuals began their first jobs and stops when they left the labor force. Thus, those who never had a job were excluded in this analysis. If subjects did not leave the labor force before age 55, the time points would be right censored when the respondents reached that age (the mandatory retirement age for women in China). As a result, we obtained 46,341 person-year records. When estimating the models, we include polynomial forms of age (up to the fifth power) to capture the nonlinear effect of duration. Moreover, as each individual would be measured at multiple time points, standard errors are adjusted to account for individual clustering effects.^5^

In the following analysis, we first estimate models without the interaction between family-related events and reform stages to show how marriage and parenthood would be associated with men and women’s job mobility patterns in general. We then include the interactions to examine how the effects of family status vary over time. Finally, we run regression models on the pooled sample with full interaction between the female dummy and all other independent variables to test the statistical difference between estimated coefficients for male and female subsamples.

## 5. Results

### 5.1 Descriptive statistics

Table 1 provides descriptive statistics for individual-level records in Panel A and person-year records in Panel B. As shown in the table, men and women differ largely in terms of mobility in both individual-level and person-year observations. Up to 21.85% of men but only 12.83% of women have ever experienced upward mobility, whereas 26.25% of men and 39.23% of women ever withdrew from the workforce at a certain life stage.

The annual upward mobility rate shows a similar pattern. In particular, after we distinguish between two types of job exits at person-year records, men and women differ in annual rate of involuntary exit (0.56 for men and 1.56 for women) but not voluntary job exit (1.10 for men and 1.09 for women). These results suggest that, compared with their male counterparts, women are less likely to experience upward mobility and more likely to leave the labor force – in particular, an involuntarily exit – due to layoff or for family reasons.

Men and women also differ in education, party membership, and hukou status. In general, compared to men, women tend to be less educated, less likely to be a Communist party member, but more likely to hold rural hukou status. On the other hand, women do not differ much from men in marital rates or having dependent children, but they have different career outcomes in terms of upward mobility and job exit rates.

**Figure 1:**
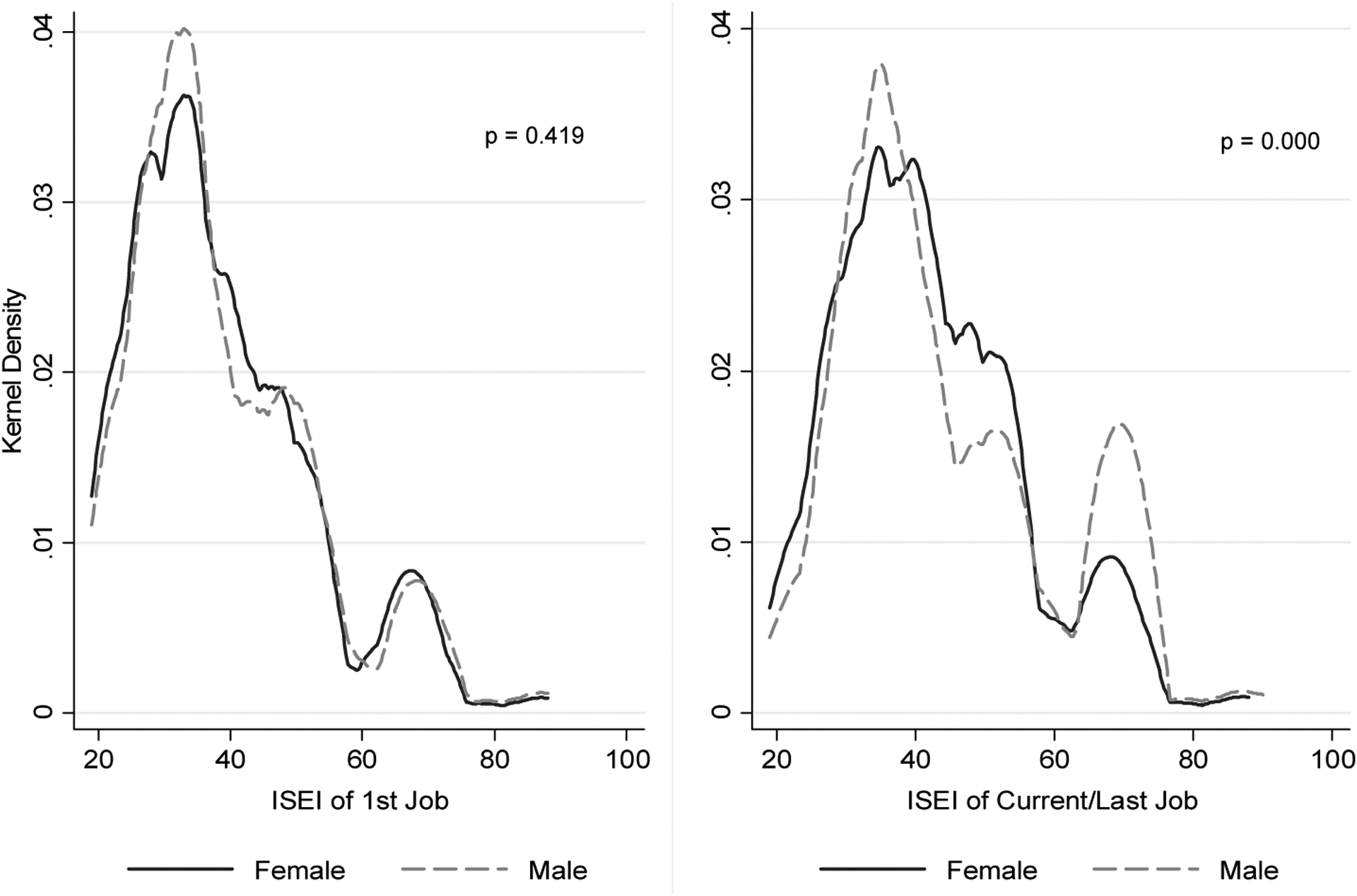
Kernel density of gender differences in ISEI of first job and current/last job, urban China *Note*: The figure is drawn based on job information from the Chinese General Social Survey in 2008. Only individuals aged 18 or above (excluding students) residing in urban areas are included.

To illustrate the divergent career paths of men and women, we also plot the kernel density graphs in Figure 1, based on the ISEI of an individual’s first occupation and current (or last) one. While the gender gap in occupational ISEI is negligible for the first occupation, women clearly lag behind in terms of occupational status in their current (or last) occupation.

**Figure 2:**
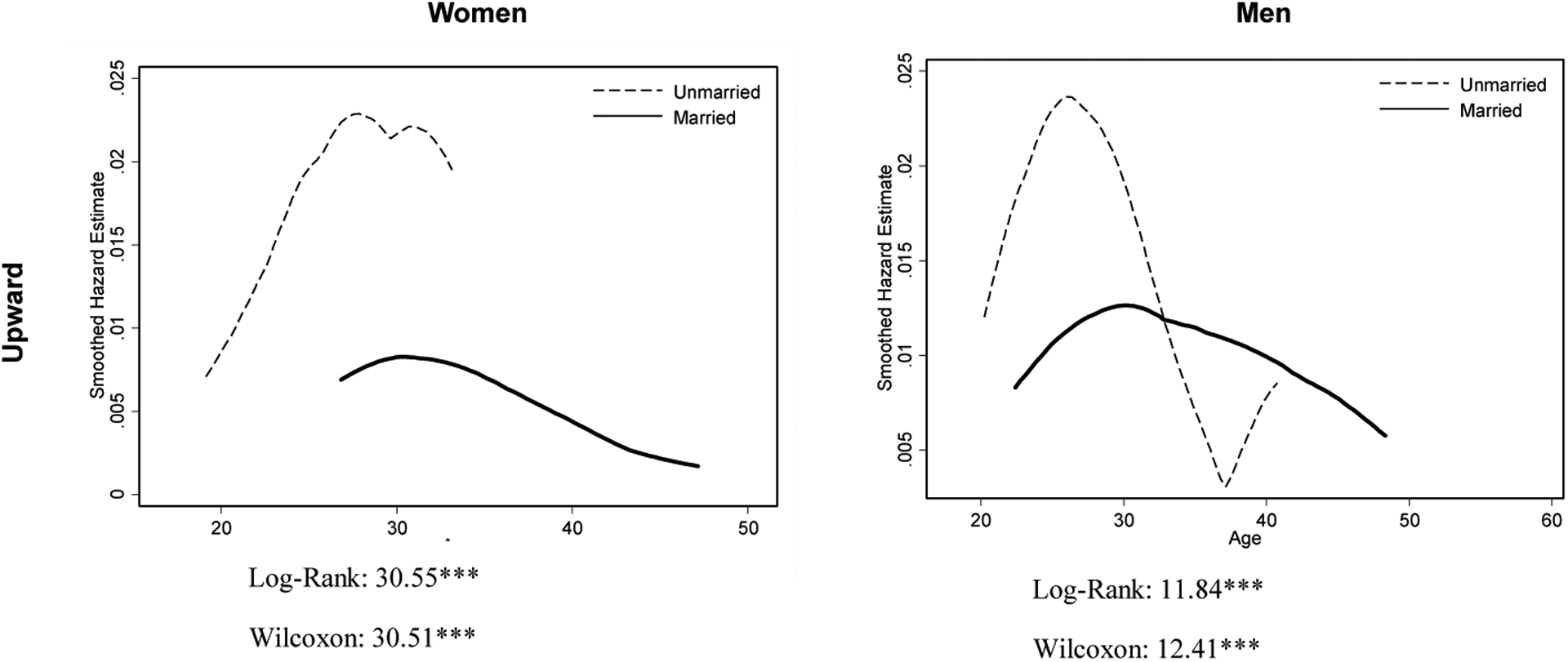

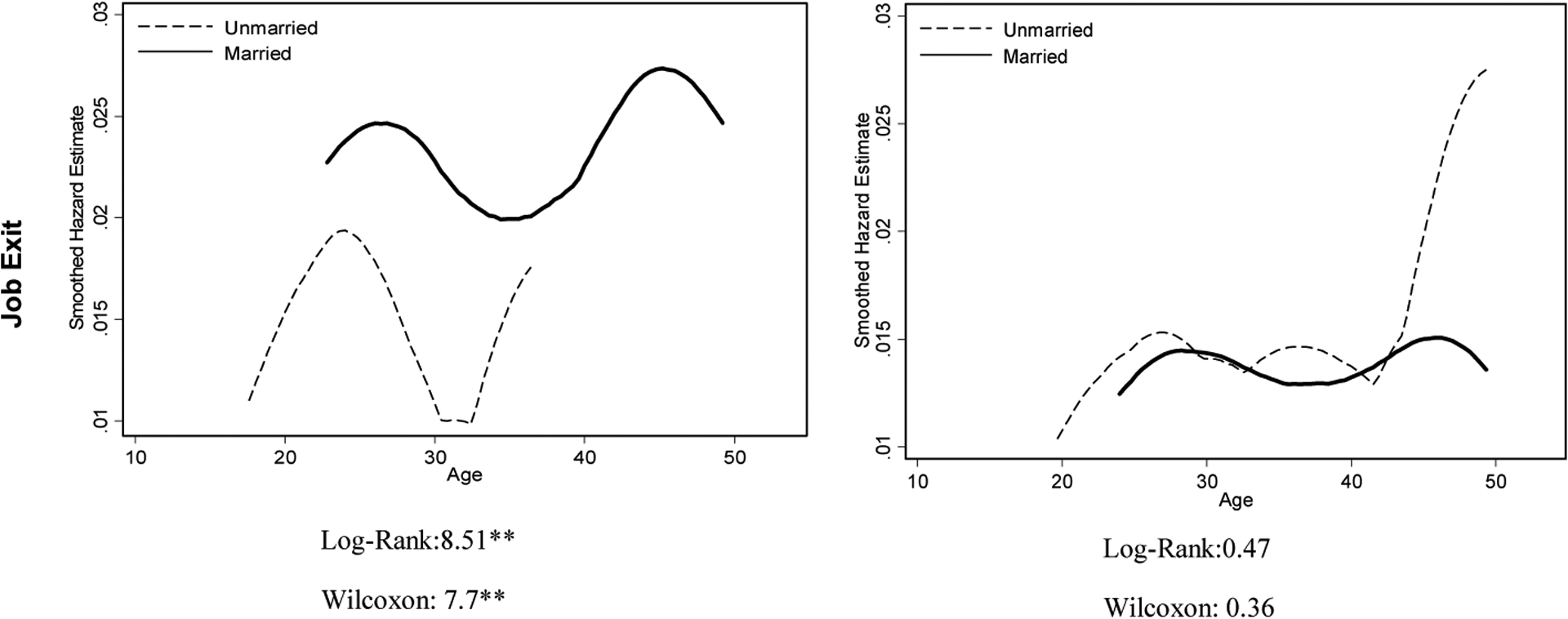
Smoothed hazard and survival function of job change by gender *Note*: The log-rank test weighs all time points equally, while the Wilcoxon test gives higher weights to earlier time points.

To further demonstrate the group difference between those married and unmarried by gender, we plot the nonparametric smoothed hazard function for upward job mobility and labor market exit in Figure 2. As seen from the results of log-rank and the Wilcoxon tests, married women are less likely than unmarried women to experience upward career mobility and more likely to withdraw from the labor force.^6^ Similar to married women, married men are also less likely to experience upward mobility. This finding seems to contradict the wage premium of fatherhood previously reported in Western societies (e.g., Killewald 2013; Loh 1996; Lundberg and Rose 2000). A plausible explanation is that lower rates of upward mobility indicate a longer time between promotions, and promotion rates are generally higher earlier in one’s career (Gibbons and Roberts 2012). As to job exit, marriage does not seem to have an impact on men’s likelihood of withdrawing from the labor force.

### 5.2 Results from event history analysis

#### 5.2.1 Marriage

Table 2 presents the results of discrete-time event history analysis on the effect of marriage on the likelihood of upward job mobility. The left two columns are baseline models for women and men (Models 1a and 2a respectively). Consistent with previous findings (Walder, Li, and Treiman 2000), education increases the likelihood of upward mobility, irrespective of gender. Party members are more likely than nonparty members to experience upward mobility. Notably, those working in the public sector are more likely to experience upward mobility than those working in the private sector, probably because the public sector in urban China has a longer career path. Holding other factors constant, married individuals are less likely than unmarried persons to achieve upward job mobility. The net odds of upward mobility for married women are only 41.2% (= 1‒ e^−0.531^) lower than those for unmarried women, whereas the net odds of upward mobility for married men are 27.0% (= 1‒e^−0.315^) lower than those for unmarried men. The confidence intervals overlap with zero for men but not for women, suggesting that marriage has effect on women but not men. Therefore, Hypothesis 1a is supported.

To demonstrate how the impact of marriage on career mobility changes over time, we include interactions between marital status and reform stages (Models 2a and 2b). For women, while the impact of marriage on the likelihood of upward mobility does not differ between the second reform stage (1993‒1998) and the early reform stage (1978‒1992), the negative coefficient of marriage increases dramatically in the late reform stage (1999‒2008). For women, the net odds of marriage on upward mobility in the late reform stage (1999‒2008) are 54.3% (=1‒e^−0.782^) lower than those in the early reform stage (1978‒1992). Hypothesis 3a is thus supported. For men, the coefficient for marriage does not seem to differ across the three reform stages.

Finally, we run regression models on the pooled sample with full interaction between gender and all other variables to test whether the effect of variables vary between men and women (Models 1c and 2c). As shown, the impact of the public sector on the likelihood of upward mobility is stronger for women than for men. This is consistent with the empirical findings about gender earnings inequality across sectors, which are employed to approximate the impact of marketization (Wu and Song 2014; He and Wu 2017).^7^ With the structural change of employment from the public to the private sectors, women have become more disadvantaged in the labor market.

The results for job exit are shown in Tables 3 and 4. Table 3 shows the result of overall job exit. As the baselines model (Model 1a and 1b) demonstrates, women are more likely than men to withdraw from the labor force upon marriage. The net odds of the job exit for married women are 2.42 times (=e ^0.882^) those of unmarried women. However, marital status does not seem to be associated with men’s job exit (Model 1b). These findings lend support to Hypothesis 1b. Models 2a and 2b further include interaction terms between marriage and reform stages. We show that, for women, the effects of marital status on job exit are more prominent in both the second reform (1993‒1998) and late reform (1999‒2008) stages than in the early reform stage (1978‒1992). For men, marriage does not seem to be associated with labor market attachment at any reform stage.

**Figure 3:**
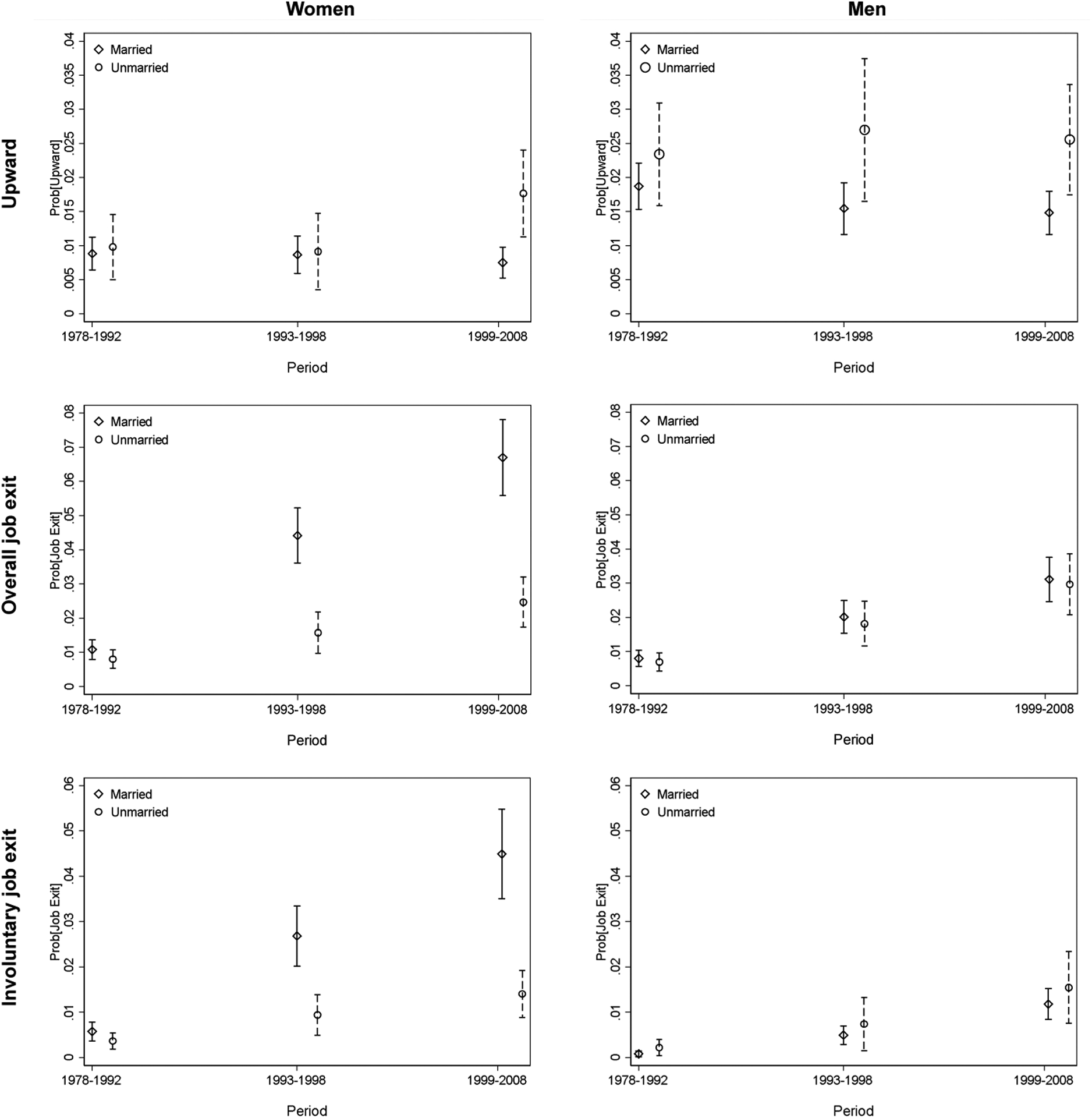
Predicted probability of job change by marital status *Note*: The figures are drawn from Models 1c and 2c in Table 2 and Models 1c and 2c in Table 3.

We further differentiate the various types of job exit and present the results in Table 4.^8^ In the baseline models (Model 1a and 1b), marriage makes women more likely to exit the job market because of layoffs or family needs. It is particularly the case in the late reform stage.^9^ However, no such pattern is found for men. Hypothesis 3a is thus supported. The gendered pattern of how transition to marriage influences individuals’ job mobility is plotted in Figure 3.

#### 5.2.2 Having dependent children

In East Asian societies, nonmarital births are rare (Raymo et al. 2015). From the life course perspective, having dependent children may exert additional pressure on women’s careers, independent of marriage (Hu and Yeung 2019; Qian and Jin 2018). In this section, we examine the impact of having dependent children of ages 0 to 6 on the likelihood of upward mobility and job exit among those who are married and test Hypotheses 1b, 2b, and 3b. The results are presented in Table 5. Further, we introduce respondents’ education relative to their spouse’s as the control considering that married women’s labor force participation is more responsive to their husbands’ employment, where relative education can be taken as a proxy for spouses’ labor market comparative advantage (Chen 2018).

In Table 5, the baseline models (Models 1a and 1b) indicate that having dependent children does not seem to be associated with upward mobility, irrespective of gender. When having dependent children is interacted with reform stages (Models 2a and 2b), for women, the effect of having dependent children does not seem to differ across reform stages. In contrast, having dependent children does not seem to affect men’s upward mobility in the early and second reform stages, but having dependent children is positively associated with career advancement in the late reform stage, suggesting the existence of a fatherhood premium in China.

**Figure 4:**
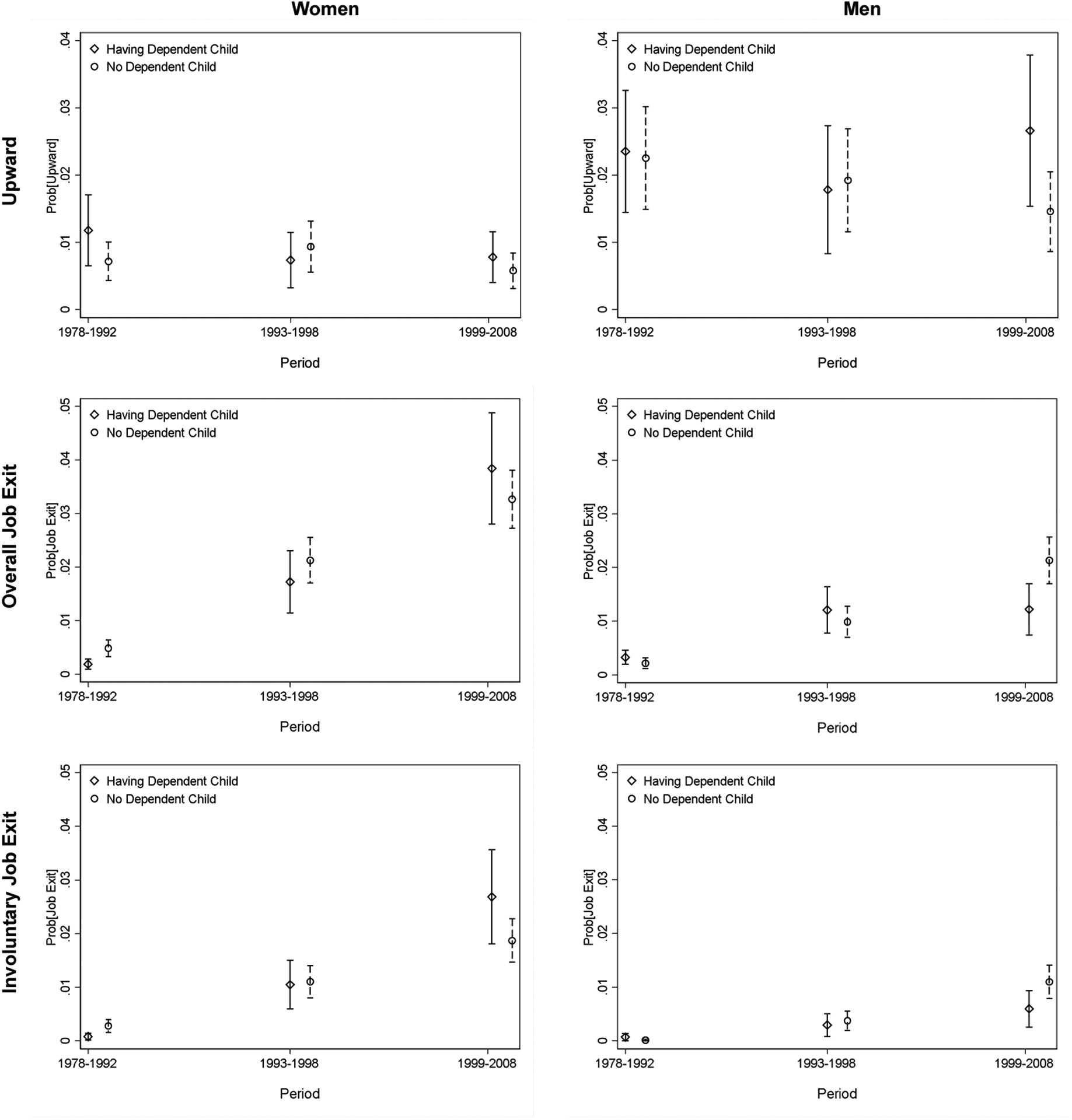
Predicted probability of job change by presence of dependent children *Note*: The figures are drawn from Models 1c and 2c in Table 4 and Model 1c and 2c in Table 5.

With regard to job exit, we also run two types of regression and present the results in Tables 6 and 7. For overall job exit, women are less likely to experience job exit when having dependent children in the early reform stage due to the work unit protection; as the reform proceeded, the disadvantage of having dependent children became more evident. As Model 2a shows, married women are more likely to leave the labor force in the late reform stage if they have dependent children. This is even more pronounced in the late reform stage (1999‒2008) than in the early reform stage (1978‒1992), as seen in Table 6. When differentiating types of job exit, we further show that such results are largely driven by involuntary job exit (Table 7). Having dependent children does not seem to affect men in the same way it does women (Model 2b in Tables 6 and 7), lending support to Hypotheses 2b and 3b. The findings are plotted in Figure 4.

## 6. Conclusions and discussions

The second half of the 20^th^ century has witnessed a steady increase in women’s education and labor force participation in Western countries, and the gender pay gap has been substantially reduced (Goldin 1990). Nevertheless, gender inequality persists in labor markets. Scholars have increasingly pointed to women’s roles in marriage and family as constraints on their career development. However, previous studies mostly focused on one relatively stable welfare regime. This study extends the research on the impact of women’s reproductive role on their career development within the context of China’s market reform when welfare regimes changed substantially, leading to the reconfiguration of the relationship between women’s paid work and family responsibilities.

We adopted the framework of two-sphere separation that originated in Western family studies. The framework was modified to incorporate historical specifics and provide a dynamic perspective on the escalating work–family conflict during China’s transition from a state to a market economy. In such a bifurcated framework, we view the pre-reform work–family arrangement under the danwei system as the private sphere relatively integrated into the public sphere, but we also view the stripping of danwei’s social responsibilities since the 1990s as the reseparation between the public and private spheres. Such a separation process provides an opportunity to understand the negative impact of marriage on women’s careers in post-danwei urban China.

Based on the retrospective CGSS 2008 data and discrete-time logit models, we found that women’s careers are more adversely affected by marriage and having dependent children than men’s careers. This pattern is more prominent in the late reform stage (1999‒2008) ‒ when the restructuring of state-owned enterprises and radical privatization eliminated much of the social functions of danwei ‒ than in earlier reform stages. The impact of marketization on women’s careers is also supported by the evidence that they are more likely to experience upward mobility and less likely to withdraw from the labor force (involuntarily, in particular) in the public sector than in the private sector, and that the sectoral differences in career outcomes are larger for women than for men.

Our findings echo but also complement earlier studies showing that marketization increases gender earnings inequality both directly through within-job discrimination and indirectly through sorting men and women into occupations with different pay (He and Wu 2017). We have charted a distinct pathway through which the withdrawal of the state affects the dynamics of gender stratification in urban China and shown how advantages/disadvantages in earnings accumulate over the life course. With the dismantling of the all-encompassing danwei after decades of economic reform, transformative social policies are called for to support working mothers, to check the rampant gender discrimination, and to promote gender equality in urban China’s labor market.

Our research design and analyses are built on the assumption that gender norms ‒ deeply rooted in the patriarchal tradition of Confucianism ‒ persist and have not been substantially undermined by state intervention in Mao’s era or even transformed through women’s improvement in education in the past decades (Ji and Wu 2018; Koo, Hui, and Pun 2020). First, this assumption, although reasonable (for the United States, see England, Levine, and Mishel 2020), is subject to empirical verification in the long run. Second, while China’s social and economic changes are a multidimensional process, we interpret the temporal trend in the role of family status on women’s career outcomes as the result of mainly marketization (in the late reform stage), without a direct measure in the models (also see footnote 4). Moreover, the current model specification, admittedly, cannot fully differentiate between age effect, period effect, and cohort effect (Yang and Land 2013). In particular, period effect may be intertwined with cohort effect. Given that the age of marriage and childbirth does not change substantially across the time span due to the enactment of nationwide family planning policy in China since early 1970s, cohort variation can be used to largely approximate period effect (Ryder 1965). In this vein, our interpretation of the result is based on the strong assumption of period effect. Finally, although the analyses of retrospective data from 2008 may have captured a historical period when structural and policy changes created penalties for women/mothers, new evidence and theoretical knowledge are needed to understand how young Chinese families have been making decisions on parenting and career development in recent years. The limitations of this article suggest the future direction of both new data collection and improved research design to gain deep understanding of gender inequality dynamics in urban China.

## Figures and Tables

**Table 1:** Descriptive statistics for selected variables (person-year), CGSS 2008

	Full sample	Men	Women
Panel A: Individual records			
Ever experienced upward mobility %	17.37	21.85	12.83
Ever exited %	32.69	26.25	39.23
Married %	82.17	82.48	81.85
Have dependent children (0–6) %	81.01	79.92	82.11
Education %			
≤Primary	13.31	11.09	15.56
Junior high	28.73	26.97	30.52
Senior high	33.65	34.38	32.91
≥College	24.31	27.56	21.01
Party member %	16.78	23.69	9.77
Rural hukou %	19.06	16.99	21.14
Public sector %	39.31	38.72	39.91
N	3,028	1,524	1,504
Panel B: Person-year records			
Job mobility rat			
Upward mobility (*100)	1.41	1.84	0.95
Exit (*100)	2.13	1.65	2.66
Involuntary exit	1.04	0.56	1.56
Voluntary exit	1.10	1.10	1.09
Reform stage			
1978–1992	42.69	42.20	43.22
1993–1999	25.96	26.05	25.86
2000–2008	31.35	31.75	30.91
Time−variant covariates			
Married %	75.50	74.07	77.05
Have dependent children (0–6) %	26.60	27.00	26.16
Party member %	15.02	21.05	8.48
Public sector %	22.90	23.66	22.08
Rural hukou %	15.35	14.71	16.04
Age	34.11	34.31	33.9
	(9.73)	(9.66)	(9.79)
Person-year	46,341	24,120	22,221

*Note*: Figures are in percentages, except for age (standard deviation shown in the parentheses).

**Table 2:** Discrete-time logit model estimating the effect of marriage on upward job mobility, CGSS 2008

	Model 1a Women	Model 1b Men	Model 1c Diff	Model 2a Women	Model 2b Men	Model 2c Diff
Marriage	−0.531 [−0.912,−0.151]	−0.315 [−0.633,0.004]	−0.217 [−0.713,0.279]	−0.135 [−0.711,0.440]	−0.136 [−0.518,0.245]	0.001 [−0.689,0.691]
Reform Stage (ref.=1978–1992)						
1993–1998	0.007 [−0.347,0.361]	−0.065 [−0.312,0.181]	0.072 [−0.359,0.503]	−0.039 [−0.791,0.713]	0.156 [−0.294,0.605]	−0.195 [−1.071,0.681]
1999–2008	0.158 [−0.189,0.504]	−0.089 [−0.321,0.144]	0.246 [−0.171,0.663]	0.663 [0.093,1.233]	0.108 [−0.289,0.505]	0.555 [−0.139,1.250]
Interaction						
Marriage*1993–1998				0.053 [−0.788,0.894]	−0.323 [−0.879,0.234]	0.375 [−0.633,1.384]
Marriage*1999–2008				−0.782 [−1.442,−0.122]	−0.283 [−0.769,0.203]	−0.499 [−1.319,0.320]
Education (ref. ≤Primary)						
Junior high	0.616 [−0.163,1.395]	0.585 [0.016,1.153]	0.032 [−0.933,0.996]	0.639 [−0.143,1.420]	0.602 [0.031,1.173]	0.037 [−0.931,1.004]
Senior high	1.002 [0.238,1.766]	1.030 [0.474,1.587]	−0.028 [−0.973,0.917]	1.025 [0.257,1.792]	1.056 [0.495,1.617]	−0.032 [−0.982,0.919]
≥College	1.483 [0.614,2.351]	1.570 [0.978,2.162]	−0.087 [−1.138,0.963]	1.520 [0.647,2.392]	1.588 [0.994,2.182]	−0.068 [−1.123,0.987]
Party member	0.845 [0.418,1.272]	0.794 [0.555,1.033]	0.052 [−0.438,0.541]	0.832 [0.407,1.257]	0.793 [0.555,1.031]	0.039 [−0.448,0.526]
Public sector	0.856 [0.572,1.139]	0.133 [−0.106,0.371]	0.723 [0.353,1.094]	0.822 [0.534,1.110]	0.119 [−0.122,0.359]	0.704 [0.328,1.079]
ISEI of 1^st^ occupation	0.005 [−0.005,0.015]	−0.000 [−0.008,0.007]	0.006 [−0.007,0.018]	0.005 [−0.005,0.015]	−0.001 [−0.008,0.007]	0.006 [−0.007,0.018]
Constant	3.611 [−53.252,60.473]	−70.175 [−129.05, −11.30]		4.796 [−50.570,60.161]	−67.877 [−126.871,−8.884]	0.001 [−0.689,0.691]
N	22,221	24,116		22,221	24,116	
Log-likelihood	−1,115.761	−2,105.437		−1,112.214	−2,104.460	
Chi-square	179.253	209.368		201.065	210.870	

*Note*: Confidence intervals are shown in brackets. In consideration of nonlinear forms of age, polynomial forms of age are added.

**Table 3:** Discrete-time logit model estimating the effect of marriage on job exit, CGSS 2008

	Model 1a Women	Model 1b Men	Model 1c Diff	Model 2a Women	Model 2b Men	Model 2c Diff
Marriage	0.882 [0.531,1.232]	0.089 [−0.257,0.435]	0.793 [0.300,1.285]	0.294 [−0.179,0.767]	0.140 [−0.384,0.665]	0.154 [−0.552,0.860]
Reform stage (ref.=1978- 1992)						
1993–1998	1.263 [1.014,1.512]	0.953 [0.671,1.234]	0.310 [−0.066,0.686]	0.722 [0.253,1.191]	0.972 [0.508,1.435]	−0.249 [−0.909,0.410]
1999–2008	1.702 [1.451,1.953]	1.421 [1.142,1.700]	0.281 [−0.094,0.656]	1.159 [0.742,1.575]	1.481 [1.040,1.923]	−0.323 [−0.930,0.284]
Interaction						
Marriage*1993–1998				0.753 [0.200,1.306]	−0.034 [−0.615,0.547]	0.787 [−0.015,1.589]
Marriage*1999–2008				0.760 [0.263,1.256]	−0.092 [−0.631,0.448]	0.851 [0.118,1.585]
Education (ref. ≤Primary)						
Junior high	−0.297 [−0.530,−0.063]	−0.152 [−0.480,0.176]	−0.144 [−0.547,0.258]	−0.310 [−0.544,−0.076]	−0.148 [−0.478,0.182]	−0.162 [−0.566,0.242]
Senior high	−0.659 [−0.915,−0.404]	−0.445 [−0.781,−0.108]	−0.215 [−0.637,0.208]	−0.676 [−0.931,−0.420]	−0.439 [−0.779,−0.099]	−0.236 [−0.662,0.189]
≥College	−1.673 [−2.119,−1.227]	−1.530 [−2.014,−1.045]	−0.143 [−0.802,0.516]	−1.688 [−2.134,−1.242]	−1.527 [−2.013,−1.042]	−0.161 [−0.820,0.499]
Party member	−0.339 [−0.825,0.146]	−0.401 [−0.746,−0.057]	0.062 [−0.534,0.657]	−0.332 [−0.819,0.155]	−0.402 [−0.747,−0.058]	0.070 [−0.526,0.667]
Public sector	0.317 [0.114,0.520]	−0.150 [−0.395,0.095]	0.467 [0.149,0.785]	0.337 [0.136,0.539]	−0.153 [−0.398,0.092]	0.490 [0.173,0.807]
ISEI of 1^st^ occupation	−0.008 [−0.015,−0.001]	−0.005 [−0.013,0.003]	−0.003 [−0.014,0.008]	−0.008 [−0.015,−0.000]	−0.005 [−0.013,0.003]	−0.003 [−0.013,0.008]
Constant	−36.272 [−69.818,−2.725]			−38.319 [−71.651,−4.987]	−43.042 [−92.105,6.022]	
*N*	22,221	24,116		22,221	24,116	
Log-likelihood	−2,469.354	−1,901.877		−2,464.298	−1,901.760	
Chi-square	473.107	242.871		464.644	246.040	

*Note*: Confidence intervals are shown in brackets. In consideration of nonlinear forms of age, polynomial forms of age and regional dummies are added.

**Table 4:** Competing risk model estimating the effect of marriage on involuntary job exit, CGSS 2008

Involuntary Exit	Model 1a Women	Model 1b Men	Model 1c Diff	Model 2a Women	Model 2b Men	Model 2c Diff
Marriage	1.052 [0.606,1.498]	−0.392 [−1.031,0.248]	1.444 [0.664,2.224]	0.455 [−0.202,1.113]	−1.011 [−2.233,0.212]	1.466 [0.078,2.853]
Reform stage (ref.=1978–1992)						
1993–1998	1.427 [1.085,1.770]	1.558 [0.926,2.190]	−0.131 [−0.849,0.588]	0.962 [0.330,1.593]	1.231 [0.279,2.183]	−0.269 [−1.411,0.872]
1999–2008	1.958 [1.621,2.294]	2.410 [1.811,3.010]	−0.453 [−1.140,0.235]	1.380 [0.812,1.948]	1.984 [1.140,2.827]	−0.604 [−1.621,0.413]
Interaction Marriage*1993–1998				0.645 [−0.104,1.394]	0.609 [−0.695,1.913]	0.037 [−1.467,1.540]
Marriage*1999–2008				0.787 [0.105,1.470]	0.738 [−0.439,1.915]	0.049 [−1.311,1.409]
Education (ref. ≤Primary) Junior high	−0.260 [−0.563,0.044]	−0.056 [−0.628,0.516]	−0.204 [−0.851,0.444]	−0.270 [−0.574,0.033]	−0.078 [−0.653,0.496]	−0.192 [−0.841,0.458]
Senior high	−0.559 [−0.885,−0.234]	−0.145 [−0.707,0.417]	−0.415 [−1.064,0.235]	−0.572 [−0.898,−0.245]	−0.174 [−0.736,0.388]	−0.398 [−1.047,0.252]
≥College	−1.696 [−2.276,−1.116]	−0.889 [−1.660,−0.119]	−0.806 [−1.771,0.158]	−1.707 [−2.287,−1.127]	−0.905 [−1.674,−0.135]	−0.803 [−1.766,0.161]
Party member	−0.698 [−1.478,0.082]	−0.672 [−1.294,−0.051]	−0.026 [−1.023,0.972]	−0.691 [−1.470,0.089]	−0.668 [−1.291,−0.044]	−0.023 [−1.021,0.975]
Public sector	0.366 [0.114,0.619]	−0.196 [−0.599,0.207]	0.563 [0.087,1.038]	0.384 [0.134,0.633]	−0.181 [−0.581,0.219]	0.564 [0.093,1.036]
ISEI of 1^st^ occupation	−0.012 [−0.022,−0.002]	−0.014 [−0.028,0.001]	0.002 [−0.016,0.019]	−0.012 [−0.021,−0.002]	−0.013 [−0.028,0.001]	0.002 [−0.016,0.019]
Constant	−75.133 [−126.42,−23.84]	−32.788 [−160.37,94.8]		−77.205 [−128.28,−26.13]	−33.660 [−160.29,92.96]	
	22,221	24,116		22,221	24,116	
*N*	−2,871.614	−2,156.864		−2,866.900	−2,155.491	
Log−likelihood	486.601	248.878		482.739	244.856	

*Note*: Confidence intervals are shown in brackets. Base category is employed. Here results for involuntary exit vs. employed are presented, whereas voluntary exit vs. employed is omitted. In consideration of nonlinear forms of age, polynomial forms of age are added.

**Table 5:** Discrete-time logit model estimating the effect of having dependent children on upward job mobility, CGSS 2008

	Model 1a Women	Model 1b Men	Model 1c Diff	Model 2a Women	Model 2b Men	Model 2c Diff
Have dependent children	0.253 [−0.168,0.675]	0.162 [−0.148,0.472]	0.191 [−0.327,0.708]	0.522 [−0.121,1.166]	0.046 [−0.338,0.429]	0.578 [−0.167,1.324]
Reform stage (ref.=1978- 1992)						
1993–1998	−0.068 [−0.486,0.351]	−0.182 [−0.503,0.139]	0.132 [−0.397,0.661]	0.279 [−0.339,0.898]	−0.166 [−0.555,0.222]	0.465 [−0.268,1.198]
1999–2008	−0.376 [−0.842,0.091]	−0.258 [−0.563,0.048]	−0.097 [−0.658,0.463]	−0.227 [−0.877,0.424]	−0.454 [−0.833,−0.076]	0.246 [−0.510,1.002]
Interaction of						
Dependent children*1993- 1998				−0.775 [−1.748,0.197]	−0.125 [−0.779,0.528]	0.004 [−0.014,0.022]
Dependent children*1999- 2008				−0.207 [−1.106,0.692]	0.585 [0.002,1.167]	−0.657 [−1.823,0.509]
Education (ref. ≤Primary)						
Junior high	0.962 [−0.071,1.995]	0.426 [−0.235,1.087]	0.466 [−0.729,1.661]	0.936 [−0.095,1.967]	0.430 [−0.230,1.090]	0.000 [0.000,0.000]
Senior high	1.251 [0.207,2.296]	0.977 [0.331,1.622]	0.285 [−0.915,1.486]	1.222 [0.183,2.261]	0.995 [0.352,1.638]	0.433 [−0.758,1.624]
≥College						
Relative education	2.238 [1.037,3.438]	1.693 [0.996,2.389]	0.549 [−0.788,1.886]	2.225 [1.033,3.418]	1.700 [1.007,2.393]	0.233 [−0.958,1.425]
(ref. Husband=Wife)						
Husband>Wife	−0.166 [−0.648,0.317]	0.005 [−0.298,0.307]	−0.141 [−0.700,0.419]	−0.159 [−0.640,0.323]	−0.006 [−0.308,0.297]	0.532 [−0.795,1.859]
Husband<Wife	−0.104 [−0.572,0.363]	0.158 [−0.298,0.615]	−0.388 [−1.052,0.276]	−0.114 [−0.582,0.355]	0.163 [−0.291,0.618]	−0.122 [−0.680,0.436]
Party member	0.784 [0.280,1.288]	0.714 [0.429,0.998]	0.070 [−0.512,0.652]	0.790 [0.282,1.297]	0.725 [0.440,1.010]	−0.394 [−1.056,0.268]
Public sector	0.481 [0.039,0.923]	0.046 [−0.292,0.384]	0.425 [−0.146,0.997]	0.497 [0.056,0.938]	0.040 [−0.297,0.377]	0.066 [−0.518,0.649]
ISEI of 1^st^ occupation	0.006 [−0.010,0.022]	0.002 [−0.007,0.012]	0.003 [−0.015,0.021]	0.006 [−0.010,0.023]	0.002 [−0.007,0.011]	0.447 [−0.123,1.018]
Constant	112.509 [−0.479,225.496]	30.104 [−51.437,111.645]		115.057 [2.981,227.132]	26.958 [−54.946,108.863]	−0.791 [−1.855,0.272]
*N*	20,547	23,262		20,547	23,262	

*Note*: Analytical sample is restricted to married person-years. Confidence intervals are shown in brackets. In consideration of nonlinear forms of age, polynomial forms of age are added.

**Table 6:** Discrete-time logit model estimating the effect of having dependent children on job exit, CGSS 2008

	Model 1a Women	Model 1b Men	Model 1c Diff	Model 2a Women	Model 2b Men	Model 2c Diff
Have dependent children	−0.154 [−0.459,0.151]	0.131 [−0.240,0.503]	−0.286 [−0.767,0.195]	−0.848 [−1.489,−0.207]	0.460 [−0.183,1.103]	−1.308 [−2.215,−0.401]
Reform stage (ref.=1978- 1992)						
1993–1998	1.943 [1.569,2.317]	1.476 [1.071,1.880]	0.467 [−0.083,1.018]	1.700 [1.256,2.143]	1.578 [1.011,2.144]	0.122 [−0.597,0.841]
1999–2008	2.589 [2.205,2.972]	2.222 [1.822,2.623]	0.366 [−0.188,0.921]	2.254 [1.802,2.705]	2.467 [1.923,3.012]	−0.213 [−0.920,0.493]
Interaction of						
Dependent children*1993–1998				0.541 [−0.225,1.306]	−0.064 [−0.850,0.723]	0.604 [−0.492,1.701]
Dependent children*1999–2008				0.981 [0.248,1.713]	−0.683 [−1.454,0.087]	1.664 [0.602,2.726]
Education (ref. ≤Primary)						
Junior high	−0.119 [−0.465,0.228]	−0.090 [−0.552,0.372]	−0.029 [−0.606,0.549]	−0.106 [−0.450,0.238]	−0.095 [−0.558,0.369]	−0.012 [−0.588,0.565]
Senior high	−0.439 [−0.818,−0.061]	−0.494 [−0.981,−0.006]	0.054 [−0.562,0.671]	−0.418 [−0.795,−0.041]	−0.508 [−0.998,−0.018]	0.090 [−0.527,0.708]
≥College	−1.416 [−2.077,−0.755]	−1.635 [−2.351,−0.918]	0.219 [−0.755,1.193]	−1.415 [−2.076,−0.754]	−1.638 [−2.355,−0.921]	0.223 [−0.751,1.197]
Relative education (ref. Husband=Wife)						
Husband>Wife	0.094 [−0.181,0.368]	0.430 [0.100,0.759]	−0.336 [−0.765,0.093]	0.082 [−0.191,0.356]	0.437 [0.107,0.767]	−0.355 [−0.784,0.074]
Husband<Wife	0.345 [0.017,0.672]	−0.240 [−0.687,0.208]	0.584 [0.030,1.138]	0.340 [0.014,0.667]	−0.241 [−0.690,0.208]	0.582 [0.027,1.136]
Party member	−0.469 [−1.037,0.099]	−0.409 [−0.839,0.020]	−0.060 [−0.771,0.652]	−0.465 [−1.030,0.100]	−0.412 [−0.843,0.020]	−0.054 [−0.764,0.657]
Public sector	0.305 [0.035,0.576]	−0.516 [−0.931,−0.100]	0.821 [0.326,1.3161	0.303 [0.033,0.572]	−0.493 [−0.908,−0.077]	0.795 [0.301,1.290]
ISEI of 1^st^occupation	−0.011 [−0.020,−0.002]	0.003 [−0.008,0.013]	−0.014 [−0.028,0.000]	−0.011 [−0.020,−0.002]	0.003 [−0.007,0.014]	−0.014 [−0.028,−0.000]
Constant	69.504 [13.365,125.643]	−40.739 [−175.034,93.555]		65.303 [9.594,121.012]	−36.478 [−170.509,97.554]	
*N*	20,547	23,262		20,547	23,262	

*Note*: Analytical sample is restricted to married person-years. Confidence intervals are shown in brackets. In consideration of nonlinear forms of age, polynomial forms of age are added.

**Table 7:** Competing risk model estimating the effect of having dependent children on involuntary job exit, CGSS 2008

Involuntary exit	Model 1a Women	Model 1b Men	Model 1c Diff	Model 2a Women	Model 2b Men	Model 2c Diff
Have dependent children	−0.215 [−0.602,0.173]	0.334 [−0.345,1.013]	−0.549 [−1.330,0.232]	−1.162 [−2.134,−0.189]	2.157 [0.368,3.946]	−3.318 [−5.354,−1.283]
Reform stage (ref.=1978–1992)						
1993–1998	2.139 [1.616,2.662]	2.141 [1.191,3.091]	−0.002 [−1.086,1.082]	1.820 [1.210,2.429]	3.372 [1.901,4.844]	−1.552 [−3.144,0.039]
1999–2008	2.975 [2.453,3.496]	3.323 [2.416,4.231]	−0.348 [−1.394,0.698]	2.565 [1.963,3.167]	4.570 [3.157,5.983]	−2.005 [−3.540,−0.470]
Interaction of						
Dependent children*1993–1998				0.815 [−0.295,1.925]	−1.877 [−3.810,0.056]	2.692 [0.464,4.920]
Dependent children*1999–2008				1.193 [0.138,2.248]	−1.995 [−3.797,−0.193]	3.188 [1.101,5.275]
Education (ref. ≤Primary)						
Junior high	−0.136 [−0.566,0.294]	−0.358 [−1.106,0.390]	0.222 [−0.641,1.084]	−0.125 [−0.553,0.303]	−0.361 [−1.109,0.386]	0.237 [−0.624,1.097]
Senior high	−0.279 [−0.739,0.180]	−0.419 [−1.172,0.334]	0.140 [−0.742,1.021]	−0.258 [−0.715,0.200]	−0.418 [−1.169,0.333]	0.160 [−0.719,1.039]
≥College	−1.559 [−2.366,−0.753]	−1.577 [−2.752,−0.402]	0.017 [−1.406,1.441]	−1.556 [−2.362,−0.750]	−1.571 [−2.744,−0.399]	0.016 [−1.406,1.438]
Relative education (ref. Husband=Wife)						
Husband>Wife	0.119 [−0.222,0.459]	0.487 [−0.054,1.028]	−0.368 [−1.007,0.270]	0.107 [−0.233,0.448]	0.494 [−0.047,1.034]	−0.386 [−1.025,0.252]
Husband<Wife	0.220 [−0.199,0.639]	−0.578 [−1.368,0.212]	0.798 [−0.096,1.692]	0.216 [−0.202,0.634]	−0.576 [−1.366,0.215]	0.792 [−0.102,1.685]
Party member	−0.742 [−1.617,0.132]	−0.685 [−1.454,0.085]	−0.057 [−1.222,1.107]	−0.740 [−1.614,0.133]	−0.690 [−1.460,0.081]	−0.051 [−1.215,1.114]
Public sector	0.425 [0.086,0.764]	−0.592 [−1.269,0.085]	1.017 [0.261,1.774]	0.425 [0.086,0.763]	−0.582 [−1.258,0.094]	1.007 [0.251,1.763]
ISEI of 1^st^ occupation	−0.014 [−0.025,−0.002]	−0.003 [−0.020,0.014]	−0.010 [−0.031,0.011]	−0.014 [−0.026,−0.002]	−0.003 [−0.020,0.014]	−0.010 [−0.031,0.010]
Constant	14.653 [−65.270,94.575]	−35.661 [−230.827,159.505]		10.032 [−68.406,88.469]	−26.297 [−242.948,190.355]	
*N*	20,547	23,262		20,547	23,262	

*Note*: Analytical sample is restricted to married person-years. Confidence intervals are shown in brackets. Base category is ‘employed.’ Here only results for ‘involuntary exit vs. employed’ are presented, whereas ‘voluntary exit vs. employed’ is omitted. In consideration of nonlinear forms of age, polynomial forms of age are added.

## Footnotes

3 The length of full-paid maternity leave increased to 90 days in 1988, according to the newly issued Female Employee Labor Protection Regulations.

4 It should be noted that there is no clear-cut difference between a layoff and a voluntary exit to take care of the family. Indeed, many women were laid off exactly because of their commitment to family and childcare. For this reason, we group them all as involuntary exits (Wu 2010).

5 Our analysis does not include reemployment, although it is important. One reason, similar to the case of demotion, is that too few individuals in our data reentered the labor force after they left. However, according to Zhang (2003), the reemployment rate in China was very low, even for those workers laid off during the restructuring of state-owned enterprises in the late 1990s. Moreover, women’s reemployment decisions were found to be less responsive to public subsidies but more responsive to family circumstances (Giles, Park, and Cai 2006). Therefore, ignoring reemployment is unlikely to lead to serious bias in our results.

6 The fluctuation in the job exit pattern over the life course (age) seems to be puzzling. For example, there is a rebound in upward mobility among unmarried women after their late 30s. We speculate that this may be due to the small sample size of unmarried women in the age group; another reason is that, in the descriptive analysis that takes age as the x-axis, the job mobility pattern reflects not only the age effect but also cohort and time effects. The main point here is to demonstrate the difference between married and unmarried men and women.

7 The three-way interactions among marital status, sector, and reform stages, however, do not differ from zero, although the sign of the coefficient is consistent with what is expected.

8 As this article aims to reveal how family roles are associated with men and women’s job exit across different reform stages, we present job exit as only the result of layoffs or family reasons versus employed to conserve space. For other types of job exit versus employed, results are available upon request.

9 It should be noted that the variations across the three reform stages may also reflect cohort difference because the young cohort was not observed in the early reform stages, but old cohorts were observed in the late reform stage (1999‒2008) at much older ages. Thus, while the estimated coefficients are more likely to reflect the de facto marriage and childbearing effect, the late stage is more likely to involve comparisons among individuals of different cohorts, providing an upper bound for the estimation.

## References

[R1] Adams M (2011). Division of household labor. In: Ritzer G and Ryan MJ (eds.). The concise encyclopedia of sociology. Malder: Wiley-Blackwell: 156–157.

[R2] Allison PD (2014). Event history and survival analysis: Regression for longitudinal event data. Thousand Oaks: SAGE Publications. doi:10.4135/9781452270029.

[R3] Becker GS (1991). A treatise on the family. Cambridge: Harvard University Press.

[R4] Beller AH (1982). Occupational segregation by sex: Determinants and changes. Journal of Human Resources 17(3): 371–392. doi:10.2307/145586.

[R5] Bian Y (1994). Work and inequality in urban China. Albany: State University of New York Press.

[R6] Bian Y and Li L (2012). The Chinese general social survey (2003‒8): Sample designs and data evaluation. Chinese Sociological Review 45(1): 70–97. doi:10.2753/CSA2162-0555450104.

[R7] Bianchi SM and Milkie MA (2010). Work and family research in the first decade of the 21st century. Journal of Marriage and Family 72(3): 705–725. doi:10.1111/j.1741-3737.2010.00726.x.

[R8] Bianchi SM, Milkie MA, Sayer LC, and Robinson JP (2000). Is anyone doing the housework? Trends in the gender division of household labor. Social Forces 79(1): 191–228. doi:10.1093/sf/79.1.191.

[R9] Blau FD and Kahn LM (2007). Changes in the labor supply behavior of married women: 1980–2000. Journal of Labor Economics 25(3): 393–438. doi:10.1086/513416.

[R10] Budig MJ, Misra J, and Boeckmann I (2012). The motherhood penalty in cross-national perspective: The importance of work–family policies and cultural attitudes. Social Politics: International Studies in Gender, State and Society 19(2): 163–193. doi:10.1093/sp/jxs006.

[R11] Cao Y and Hu CY (2007). Gender and job mobility in post-socialist China: A longitudinal study of job changes in six coastal cities. Social Forces 85(4): 1535–1560. doi:10.1353/sof.2007.0065.

[R12] Cha Y (2010). Reinforcing separate spheres: The effect of spousal overwork on men’s and women’s employment in dual-earner households. American Sociological Review 75(2): 303–329. doi:10.1177/0003122410365307.

[R13] Cha Y (2013). Overwork and the persistence of gender segregation in occupations. Gender and Society 27(2): 158–184. doi:10.1177/0891243212470510.

[R14] Chen M (2018). Does marrying well count more than career? Personal achievement, marriage, and happiness of married women in urban China. Chinese Sociological Review 50(3): 240–274. doi:10.1080/21620555.2018.1435265.

[R15] Cheng S (2016). The accumulation of (dis)-advantage: The intersection of gender and race in the long-term age effect of marriage. American Sociological Review 81(1): 29–56. doi:10.1177/0003122415621263.

[R16] Connell RW (1985). Theorizing gender. Sociology 19(2): 260–272. doi:10.1177/0038038585019002008.

[R17] Cook S and Dong XY (2011). Harsh choices: Chinese women’s paid work and unpaid care responsibilities under economic reform. Development and Change 42(4): 947–965. doi:10.1111/j.1467-7660.2011.01721.x. 22164881

[R18] Costa DL (2000). From mill town to board room: The rise of women’s paid labor. Journal of Economic Perspectives 14(4): 101–122. doi:10.1257/jep.14.4.101.

[R19] Cotter DA, Hermsen JM, and Vanneman R (1999). Systems of gender, race, and class inequality: Multilevel analyses. Social Forces 78(2): 433–640. doi: 10.2307/3005563.

[R20] Edwards OLF (2001). Gender and changing role of women. In: Barney M and Malder MA (eds.). A companion to 19^th^ century America. Malder: Wiley-Blackwell: 225‒226.

[R21] Engels F (1978 [1891]). The origin of the family, private property and the state (excerpt). In: Tucker RC (ed.). The Marx-Engels reader. New York: Norton: 734–759.

[R22] England P, Levine A, and Mishel E (2020). Progress toward gender equality in the United States has slowed or stalled. Proceeding of National Academy of Sciences 117(13): 6990‒6997. doi:10.1073/pnas.1918891117. PMC7132302 32229559

[R23] Esping-Anderson G (1999). Social foundations of postindustrial economies. Oxford: Oxford University Press. doi:10.1093/0198742002.001.0001.

[R24] Fernandez RM and Friedrich C (2011). Gender sorting at the application interface. Industrial Relations 50(4): 591–609. doi:10.1111/j.1468-232X.2011.00654.x.

[R25] Ferree MM (1990). Beyond separate spheres: Feminism and family research. Journal of Marriage and Family 52(4): 865–883. doi:10.2307/353307.

[R26] Fullerton H (1999). Labor force participation: 75 years of change, 1950–98 and 1998–2025. Monthly Labor Review 1999(December): 3–12.

[R27] Ganzeboom HBG and Treiman DJ (1996). Internationally comparable measures of occupational status for the 1988 international standard Classification of Occupations. Social Science Research 25(3): 201–239. doi:10.1006/ssre.1996.0010.

[R28] Gibbons RS and Roberts J (2012). The handbook of organizational economics. Princeton: Princeton University Press.

[R29] Giles J, Park A, and Cai F (2006). Re-employment of dislocated workers in urban China: The roles of information and incentives. Journal of Comparative Economics 34(3): 582–607. doi:10.1016/j.jce.2006.06.006.

[R30] Goldin C (1990). Understanding the gender gap. New York: Oxford University Press.

[R31] Gornick JC and Jacobs JA (1998). Gender, the welfare state, and public employment: A comparative study of seven industrialized countries (in Comparative Studies of the Welfare State). American Sociological Review 63(5): 688–710. doi:10.2307/2657334.

[R32] Gornick JC and Meyers MK (2003). Families that work: policies for reconciling parenthood and employment. New York: The Russell Sage Foundation.

[R33] Government Administration Council of the Central People’s Government. (1951, 10 1). Decision to change the education system. Retrieved October 10, 2014, from http://www.reformdata.org/content/20130222/17235.html.

[R34] Guo J and Xiao SW (2013). Through the gender lens: A comparison of family policy in Sweden and China. China Journal of Social Work 6(3): 228‒243. doi:10.1080/17525098.2013.840663.

[R35] Gupta S (1999). The effects of transitions in marital status on men’s performance of housework. Journal of Marriage and Family 61(3): 700‒711. doi:10.2307/353571.

[R36] Hausmann R, Tyson LD, and Zahidi S (2009). The global gender gap report 2009. http://www3.weforum.org/docs/WEF_GenderGap_Report_2009.pdf.

[R37] He G and Wu X (2017). Marketization, occupational segregation, and gender earnings inequality in urban China. Social Science Research 65: 96–111. doi:10.1016/j.ssresearch.2016.12.001. 28599783

[R38] He G and Wu X (2018). Dynamics of the gender earnings inequality in reform-era urban China. Work, Employment and Society 32(4): 726–746. doi:10.1177/0950017017746907.

[R39] He G and Wu X (2019). Foreign domestic helpers hiring and women’s labor supply in Hong Kong. Chinese Sociological Review 51(4): 397‒420. doi:10.1080/21620555.2019.1630814.

[R40] He G and Zhou M (2018). Gender difference in early occupational attainment: The roles of study field, gender norms, and gender attitudes. Chinese Sociological Review 50(3): 339–366. doi:10.1080/21620555.2018.1430509.

[R41] Hegewisch A and Gornick JC (2011). The impact of work-family policies on women’s employment: A review of research from OECD countries. Community, Work and Family 14(2): 119–138. doi:10.1080/13668803.2011.571395.

[R42] Hobson B (2006). The evolution of the women-friendly state: Opportunities and constraints in the Swedish welfare state. In: Razavi S and Hassim S (eds). Gender and social policy in a global context. Social Policy in a Development Context London: Palgrave Macmillan: 151‒172. doi:10.1057/9780230625280_7.

[R43] Hout M and DiPrete TA (2006). What we have learned: RC28’s contribution to knowledge about social stratification. Research in Social Stratification and Mobility 24(1): 1–20. doi:10.1016/j.rssm.2005.10.001.

[R44] Hu S and Yeung WJ (2019). Education and childrearing in East Asia. Chinese Sociological Review 51(1): 29–56. doi:10.1080/21620555.2019.1571903.

[R45] Hughes J and Maurer-Fazio M (2002). Effects of marriage, education and occupation on the female /male wage gap in China. Pacific Economic Review 7(1): 137–156. doi:10.1111/1468-0106.00156.

[R46] Jacobs JA (1989). Long-term trends in occupational segregation by sex. American Journal of Sociology 95(1): 160–173. doi:10.1086/229217.

[R47] Ji Y and Wu X (2018). New gender dynamics in post-reform China: Family, education, and labor market. Chinese Sociological Review 50(3): 231–239. doi:10.1080/21620555.2018.1452609.

[R48] Ji Y, Wu X, Sun S, and He G (2017). Unequal care, unequal work: Toward a more comprehensive understanding of gender inequality in post-reform urban China. Sex Roles 77: 765–778. doi:10.1007/s11199-017-0751-1.

[R49] Juhn C and Potter S (2006). Changes in labor force participation in the United States. The Journal of Economic Perspectives 20(3): 27–46. doi:10.1257/jep.20.3.27.

[R50] Kan MY and He G (2018). Resource bargaining and gender display in housework and care work in modern China. Chinese Sociological Review 50(2): 188–230. doi:10.1080/21620555.2018.1430506.

[R51] Killewald A (2013). A reconsideration of the fatherhood premium: Marriage, residence, biology, and the wages of fathers. American Sociological Review 78(1): 96–116. doi:10.1177/0003122412469204.

[R52] Kolberg JE and Esping-Andersen G (1991). Welfare states and employment regimes? International Journal of Sociology 21(1 The welfare state as employer): 3–35. doi:10.1080/15579336.1991.11770006.

[R53] Koo A, Hui BPH, and Pun N (2020). Gender ideologies of youth in post-socialist China: Their gender-role attitudes, antecedents, and socio-psychological impacts. Chinese Sociological Review 52(5): 487–514. doi:10.1080/21620555.2020.1768366.

[R54] Levanon A and Grusky DB (2016). The persistence of extreme gender segregation in the twenty-first century. American Journal of Sociology 122(2): 573–619. doi:10.1086/688628.

[R55] Loh ES (1996). Productivity differences and the marriage premium for white males. The Journal of Human Resources 31(3): 566–589. doi:10.2307/146266.

[R56] Lopata H and Thorne B (1978). On the term ‘sex roles’. Signs 3(3): 718–721. doi:10.1086/493523.

[R57] Lundberg S and Rose E (2000). Parenthood and the earnings of married men and women. Labor Economics 7(6): 689–710. doi:10.1016/S0927-5371(00)00020-8.

[R58] Mandel H (2010). Winners and losers: The consequences of welfare state policies for gender wage inequality. (LIS Working Paper Series 550). Luxembourg: Luxembourg Income Study (LIS). doi:10.1093/esr/jcq061.

[R59] Mandel H and Semyonov M (2006). A welfare state paradox: State interventions and women’s employment opportunities in 22 countries. American Journal of Sociology 111(6): 1910–1949. doi:10.1086/499912.

[R60] Marini MM (1989). Sex differences in earnings in the United States. Annual Review of Sociology 15(1): 343–380. doi:10.1146/annurev.so.15.080189.002015.

[R61] Ministry of Education (2007). Educational statistical yearbook of China. Beijing: China Statistics Press.

[R62] Mu Z and Xie Y (2016). ‘Motherhood penalty’ and ‘fatherhood premium’? Fertility effects on parents in China. Demographic Research 35(47): 1373–1410. doi:10.4054/DemRes.2016.35.47. 30568537 PMC6296818

[R63] Naughton B (2006). The Chinese economy: Transitions and growth. Boston: MIT Press.

[R64] Orloff AS (1993). Gender and the social rights of citizenship: The comparative analysis of gender relations and welfare states. American Sociological Review 58(3): 303–328. doi:10.2307/2095903.

[R65] Orloff AS (1996). Gender in the welfare state. Annual Review of Sociology 22: 51–78. doi:10.1146/annurev.soc.22.1.51.

[R66] Orloff AS (2002). Explaining US welfare reform: Power, gender, race and the US policy legacy. Critical Social Policy 22(1): 96–118. doi:10.1177/02610183020220010801.

[R67] Parish W and Busse S (2000). Gender and work. In: Tang W and Parish W (eds.). Chinese urban life under reform. Cambridge: Cambridge University Press: 209‒231.

[R68] Qian Y and Jin Y (2018). Women’s fertility autonomy in urban China: The role of couple dynamics under the universal two-child policy. Chinese Sociological Review 50(3): 275‒309. doi:10.1080/21620555.2018.1428895.

[R69] Raymo JM, Park H, Xie Y, and Yeung WJJ (2015). Marriage and family in east Asia: Continuity and change. Annual Review of Sociology 41(1): 471–492. doi:10.1146/annurev-soc-073014-112428. PMC6070151 30078932

[R70] Reskin B (1993). Sex segregation in the Workplace. Annual Review of Sociology 19(1): 241–270. doi:10.1146/annurev.so.19.080193.001325.

[R71] Ryder NB (1965). The cohort as a concept in the study of social change. American Sociological Review 30(6): 843‒861. doi:10.2307/2090964. 5846306

[R72] Sandberg S (2013). Lean in: Women, work, and the will to lead. New York: Random House.

[R73] Shavit Y and Blossfeld HP (eds.) (1993). Persistent inequality: Changing educational attainment in thirteen countries. Boulder: Westview Press.

[R74] Slaughter AM (2012). Why women still can’t have it all. The Atlantic (July/August): 85–102.

[R75] Song S (2011). The private embedded in the public: The state’s discourse on domestic work, 1949–1966. Research on Women in Modern Chinese History 19: 131–172 [in Chinese].

[R76] State Administration Council of People’s Republic of China (1951). Labor insurance regulations of the People’s Republic of China (Order No. 134 of 1951), February 1951.

[R77] Stone P (2007). Opting out? Why women really quit careers and head home. Berkeley: University of California Press. doi:10.1525/9780520941793.

[R78] Sun S and Chen F (2017). Women’s employment trajectories during early adulthood in urban China: A cohort comparison. Social Science Research 68: 43–58. doi:10.1016/j.ssresearch.2017.09.005. 29108599

[R79] Treiman DJ and Hartmann HI (eds.) (1981). Women, work, and wages: Equal pay for jobs of equal value. Washington, D.C.: National Academy Press.

[R80] Van der Lippe T and Van Dijk L (2002). Comparative research on women’s employment. Annual Review of Sociology 28(1): 221–241. doi:10.1146/annurev.soc.28.110601.140833.

[R81] Walder AG (1986). Communist neo-traditionalism: Work and authority in Chinese industry. Berkeley: University of California Press.

[R82] Walder AG (1992). Property rights and stratification in socialist redistributive economies. American Sociological Review 57(4): 524‒539. doi:10.2307/2096099.

[R83] Walder AG, Li B, and Treiman DJ (2000). Politics and life chances in a state socialist regime: Dual career paths into the urban Chinese elite, 1949 to 1996. American Sociological Review 65(2): 191–209. doi:10.2307/2657437.

[R84] Whyte MK and Parish WL (1984). Urban life in contemporary China. Chicago: University of Chicago Press.

[R85] Wolf M (1985). Revolution postponed [sound recording]: Women in contemporary China. Stanford: Stanford University Press.

[R86] Wu X (2002). Work units and income inequality: The effect of market transition in urban China. Social Forces 80(3): 1069–1099. doi:10.1353/sof.2002.0013.

[R87] Wu X (2010). Voluntary and involuntary job mobility and earnings inequality in urban China, 1993‒2000. Social Science Research 39(3): 382–395. doi:10.1016/j.ssresearch.2009.11.003.

[R88] Wu X (2019). Inequality and social stratification in post-socialist China. Annual Review of Sociology 45: 363–382. doi:10.1146/annurev-soc-073018-022516.

[R89] Wu X and Song X (2014). Ethnic stratification amid China’s economic transition: Evidence from the Xinjiang Uyghur Autonomous Region. Social Science Research 44: 158‒172. doi:10.1016/j.ssresearch.2013.12.002. 24468441

[R90] Wu X and Treiman DJ (2007). Inequality and equality under Chinese socialism: The hukou system and intergenerational occupational mobility. American Journal of Sociology 113(2): 415–445. doi:10.1086/518905.

[R91] Wu X, Ye H, and He G (2014). Fertility decline and women’s status improvement in China. Chinese Sociological Review 46(3): 3–25. doi:10.2753/CSA2162-0555460301.

[R92] Wu Y and Zhou D (2015). Women’s labor force participation in urban China, 1990–2010. Chinese Sociological Review 47(4): 314–342. doi:10.1080/21620555.2015.1036234.

[R93] Xu D and Wu X (2021). From political power to personal wealth: Privatization and elite opportunity in post-reform China. Journal of Contemporary China in press

[R94] Yang Y and Land KC (2013). Age-period-cohort analysis: New models, methods, and empirical applications. CRC Press. doi:10.1201/CHINTSTASER.

[R95] Yeh AG, Yang FF, and Wang J (2015). Economic transition and urban transformation of China: The interplay of the state and the market. Urban Studies 52(15): 2822–2828.

[R96] Zhang J (2003). Urban xiagang, unemployment and social support policies: A literature review of labor market policies in transitional China. Report to the World Bank, Washington, DC.

[R97] Zhang Y and Hannum E (2015). Diverging fortunes: The evolution of gender wage gaps for singles, couples, and parents in China. Chinese Journal of Sociology 1(1): 15–55. doi:10.1177/2057150X14568769.

[R98] Zhang Y, Hannum E, and Wang M (2008). Gender-based employment and income differences in urban China: Considering the contributions of marriage and parenthood. Social Forces 86(4): 1529–1560. doi:10.1353/sof.0.0035.

[R99] Zhao M and Hannum E (2019). Stark choices: Work-family tradeoffs among migrant women and men in urban China. Chinese Sociological Review 51(4): 365–396. doi:10.1080/21620555.2019.1635879. PMC8238465 34188970

[R100] Zhou X, Tuma NB, and Moen P (1997). Institutional change and patterns of job shifts in urban China: 1949 to 1994. American Journal of Sociology 62(3): 339–365. doi:10.2307/2657310.

[R101] Zuo J and Bian Y (2001). Gendered resources, division of housework, and perceived fairness ‒ A case in urban China. Journal of Marriage and Family 63(4): 1122‒ 1133. doi:10.1111/j.1741-3737.2001.01122.x.

[R102] Zuo J and Jiang Y (2009). Urban women’s work and family in social transition. Beijing: Contemporary China Publishing House.

